# Serum homocysteine levels predict poor recovery and relapse in first-attack myelin oligodendrocyte glycoprotein antibody disease

**DOI:** 10.3389/fneur.2025.1619479

**Published:** 2025-07-28

**Authors:** Yanfei Li, Yanjie Jia

**Affiliations:** Department of Neurology, The First Affiliated Hospital of Zhengzhou University, Zhengzhou, China

**Keywords:** homocysteine, first-attack myelin oligodendrocyte glycoprotein antibody disease, predictor, relapse, recovery

## Abstract

**Background and objective:**

Myelin oligodendrocyte glycoprotein antibody disease (MOGAD) is an inflammatory demyelinating disease with a high risk of recurrence and progressive disability, and it is crucial to find sensitive and reliable biomarkers for prognosis and the early prediction of relapse. In the study we investigated whether serum homocysteine (Hcy) levels are associated with a poor prognosis or risk of relapse in patients with first-attack MOGAD.

**Methods:**

We enrolled patients diagnosed as having first-attack MOGAD between January 2019 and December 2024 in this retrospective study. Clinical data and initial Expanded Disability Status Scale (EDSS) scores were collected and analyzed. Clinical outcomes were measured using the final EDSS score and relapse events. We used logistic regression models and Cox regression analysis to determine the association between Hcy levels and clinical recovery and relapse.

**Results:**

Seventy patients (female, *n* = 36; male, *n* = 34) with first-attack MOGAD were included in this study. The final EDSS scores (*p* = 0.015) and relapse rates (*p* = 0.039) were higher in the high Hcy group than in the normal Hcy group. Multivariate analysis results indicated that Hcy levels [odds ratio (OR) 1.126; 95% confidence interval (CI) 1.005–1.261, *p* = 0.04] and the initial EDSS scores (OR 2.017, 95% CI 1.266–3.214, *p* = 0.003) were independent risk factors for predicting poor recovery. Kaplan–Meier survival analysis showed that Hcy levels were a predictor of relapse in patients with MOGAD (log-rank test *p* = 0.029). The results of the multivariate Cox proportional hazards model indicated that Hcy levels [hazard ratio (HR) 1.088, 95% CI 1.020–1.161, *p* = 0.011] were related to MOGAD relapse.

**Conclusion:**

We identified Hcy levels as an independent risk factor for predicting poor clinical recovery in patients with first-attack MOGAD. Hcy levels were also significantly associated with the relapse of MOGAD.

## 1 Introduction

Myelin oligodendrocyte glycoprotein antibody disease (MOGAD) is an autoimmune inflammatory demyelinating disorder of the central nervous system (CNS) characterized by the presence of myelin oligodendrocyte glycoprotein (MOG) antibodies (1). MOG is a component of the CNS myelin sheath localized on the outermost layer of the oligodendrocyte, where it plays a critical role in the regulation of oligodendrocyte microtubule stability and adhesion of myelin fibers. MOG antibodies can induce myelin sheath and oligodendrocyte destruction, leading to inflammatory demyelination of the CNS (2). The majority of patients with MOGAD present with acute disseminated encephalomyelitis (ADEM), transverse myelitis (TM), recurrent optic neuritis (ON), neuromyelitis optica spectrum disorders (NMOSD) and cortical encephalitis. The disease course of MOGAD can be either monophasic or relapsing. A relapsing event disease course has been reported in 44–83% of patients, with residual disability due to relapse in 50–80% of these patients (3, 4). As many patients experience the adverse consequences of relapse and residual disability due to MOGAD, identification of reliable and sensitive biomarkers for predicting the prognosis of MOGAD is necessary. The presence of such biomarkers can indicate when proactive measures should be taken to prevent relapse and improve the prognosis of MOGAD.

Homocysteine (Hcy) is a nonessential sulfur-containing amino acid, derived from methionine metabolism, that depends on levels of maintained folate, vitamin B12, and vitamin B6 (5). Elevated Hcy levels can cause oxidative stress and mitochondrial dysfunction by increasing reactive oxygen species production and can promote excitotoxicity via stimulation of N-methyl-D-aspartate receptors (NMDARs), which induces neuronal injury and apoptosis (6–8). Previous studies showed that MOGAD may be more likely than NMOSD to co-exist with anti-NMDAR encephalitis (9).

Studies associating serum Hcy levels with the prognosis of multiple sclerosis (MS) and neuromyelitis optica spectrum disorders (NMOSDs) have been reported (10, 11); however, few studies have reported on the potential role of Hcy in predicting the prognosis and relapse of MOGAD (12). As MOGAD, NOMSD and MS are inflammatory demyelinating diseases of the central nervous system, therefore we investigated the possible association between Hcy levels and prognosis and relapse risk of first-attack MOGAD in the study.

## 2 Materials and methods

### 2.1 Participants

We retrospectively enrolled patients diagnosed with MOGAD at the First Affiliated Hospital of Zhengzhou University from January 2019 to December 2024 in this study. MOGAD was diagnosed based on the 2023 International Consensus Diagnostic Criteria for MOGAD (13).

The inclusion criteria were as follows: (1) patients seropositive for MOG antibodies, as detected by a live cell-based assay (CBA) using HEK293 cells expressing full-length human MOG with a secondary antibody to human IgG1 and (2) patients with confirmed first-attack MOGAD.

**Figure 1:**
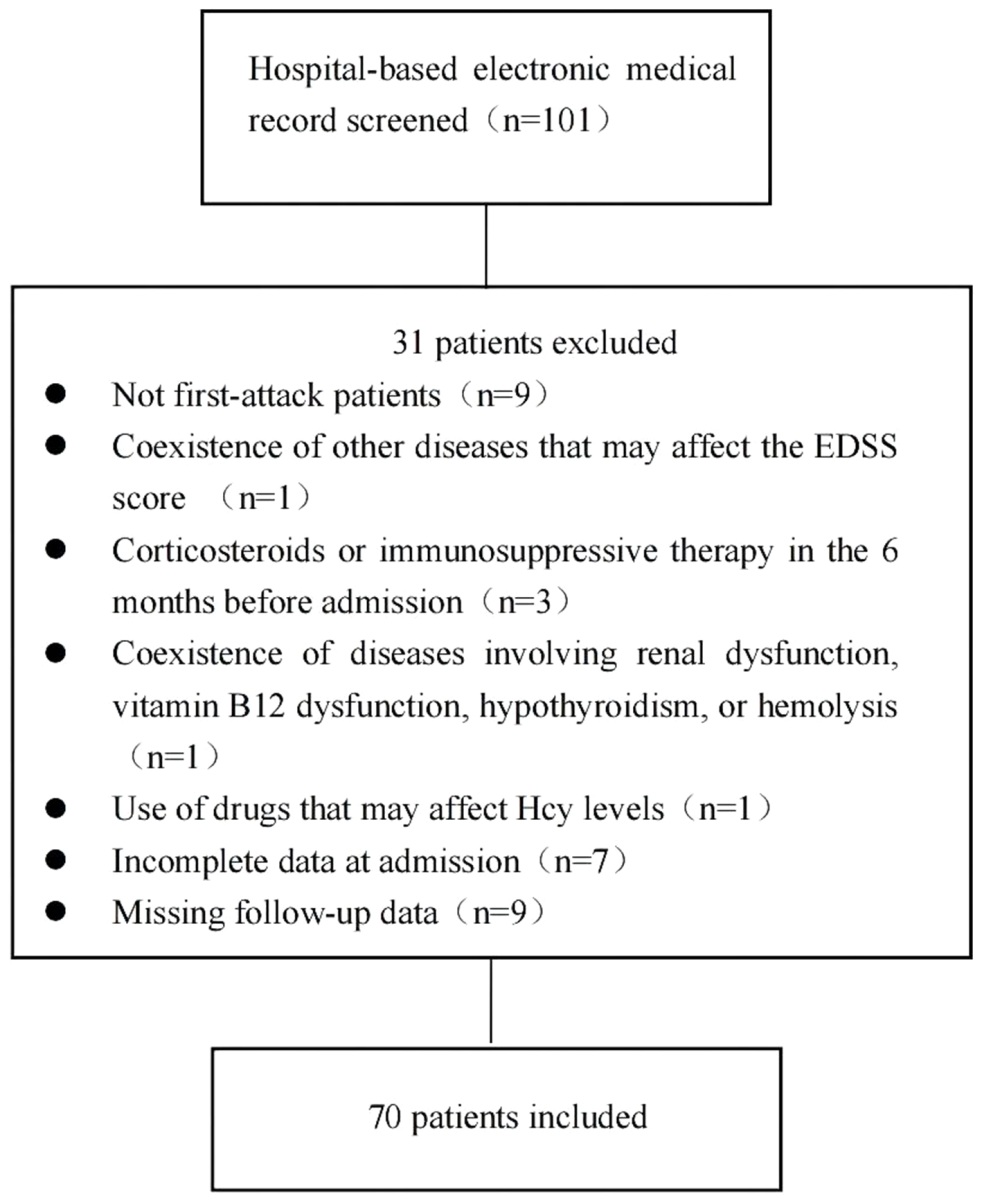
Overview of the patient selection process.

The exclusion criteria were as follows: (1) coexistence of other diseases that may affect the Expanded Disability Status Scale (EDSS) score; (2) coexistence of diseases involving renal dysfunction, vitamin B12 dysfunction, hypothyroidism, or hemolysis; (3) treatment with an Hcy-lowering drug before admission; (4) use of drugs affecting Hcy levels, such as isoniazid, phenytoin, levodopa, medroxyprogesterone, and others, before admission (14); (5) use of corticosteroids or immunosuppressive therapies in the 6 months before admission; (6) incomplete data; and (7) missing follow-up data. The detailed selection process is shown in Figure 1.

This study was approved by the Ethics Committee of the First Affiliated Hospital of Zhengzhou University (2023-KY-0398) and was conducted according to the principles of the Declaration of Helsinki.

### 2.2 Data collection and treatment

Clinical data, including age at disease onset, sex, comorbidities, treatments, laboratory test results, and cerebrospinal fluid (CSF) analysis results at admission, were collected. The EDSS scores at admission and follow-up were set as the initial EDSS scores and final EDSS scores, respectively, and were evaluated by an experienced neurologist according to the method previously reported (15). Follow-up data were obtained via annual clinic visits or telephone interviews every 3 months. The last follow-up date was March 31, 2025.

Blood samples were collected from patients after an overnight fasting period (from 7:00 am to 8:00 am the next day and before the administration of any treatments). Patients were tested for MOG antibodies using a cell-based assay. Hcy levels were detected by an automated chemistry analyzer (Roche Group, Sweden). Hyperhomocysteinemia (high hcy group) was defined as an Hcy concentration >15 μmol/L (16, 17). Serum folic acid and vitamin B12 levels were detected by chemiluminescent immunoassay on the Maglumi 2000 Plus analyzer (Shenzhen New Industries Biomedical Engineering Company Limited, China). Tests were conducted in accordance with the manufacturer’s protocols; the examiners were blinded to the patients’ diagnoses and clinical symptoms.

### 2.3 Imaging data

MRI scans were performed using a 3.0 T scanner (Philips Healthcare, Amsterdam, Netherlands). Sagittal T1-weighted images (T1WIs); axial T1WIs; T2-weighted images (T2WIs); axial/sagittal fast fluid-attenuated inversion recovery images; axial diffusion weighted images; apparent diffusion coefficient mapped images; contrast-enhanced axial, coronal, and sagittal T1WIs of the brain were analyzed. And sagittal T1WIs, sagittal T2WIs, axial T1WIs, and axial T2WIs of the spine were analyzed. Gadolinium glutamine was injected as the contrast agent.

### 2.4 Clinical outcomes

The clinical outcomes of the study were disability—according to the final EDSS score—and relapse event—at the last follow-up. The final EDSS score was converted into categorical variables. To explore the association between Hcy levels and clinical outcomes, patients who exhibited different final EDSS scores were divided into two groups. Patients with an EDSS score >3 were defined as having a poor recovery, while those with an EDSS score ≤3 were defined as having a good recovery. Relapse was defined as the presence of new-onset or recurrent neurological symptoms lasting for ≥24 h and resulting in an increase in the EDSS score of ≥0.5 points from the patient’s lowest score.

### 2.5 Statistical analyses

Normally distributed, continuous data are presented as mean±standard deviation. Continuous data with a non-normal distribution are presented as median (interquartile range). The Kolmogorov–Smirnov test was used to test the normality of the continuous data. Categorical variables are expressed as frequencies (percentages, %). The differences between groups were analyzed using the Student’s t-test and Wilcoxon test, for normally and non-normally distributed data, respectively. Categorical data were compared using the chi-square test when comparing numbers ≥5 or Fisher’s exact test when comparing numbers <5. Univariate logistic regression analysis was used to screen factors that might affect recovery in first-attack MOGAD. Variables with a significance level of *p* < 0.1 in the univariate logistic regression analysis were included in the basic multivariate logistic regression analysis model. Variables with *p* < 0.1 in the univariate logistic regression analysis or variables that may have had an impact on the final EDSS scores (including age at onset, sex, and initial EDSS scores) and factors that could affect Hcy levels (including levels of folic acid and vitamin B12) were included in the adjusted multivariate logistic regression analysis model to analyze the independent effects of Hcy levels on recovery. The correlation between Hcy levels and final EDSS scores was analyzed using Spearman’s correlation analysis. Kaplan–Meier analysis was performed to analyze the effect of Hcy levels on the timing of MOGAD relapse. A univariate Cox proportional hazards model was used to screen variables that might affect relapse in first-attack MOGAD. Variables with a significance level of *p* < 0.1 in the univariate Cox regression analysis were included in the basic multivariate Cox regression analysis model. Variables with *p* < 0.1 in the univariate Cox regression analysis or variables that may have had an impact on relapse (including age at onset, sex, and initial EDSS scores) and factors that could affect Hcy levels (including levels of folic acid and vitamin B12) were included in the adjusted multivariate Cox regression analysis model to analyze the independent effects of Hcy levels on relapse.

All statistical analyses were performed using SPSS (version 26.0; IBM, Armonk, NY, USA) and the diagram was generated with GraphPad Prism 8. Statistical significance was set at *p* < 0.05.

## 3 Results

### 3.1 Demographics and clinical characteristics of patients

After inspecting the database of the First Affiliated Hospital of Zhengzhou University between January 2019 and December 2024, 101 patients were diagnosed as having MOGAD, with 70 patients meeting the inclusion criteria. Patients were divided into two groups according to serum Hcy levels: the high Hcy group (serum Hcy levels >15 μmol/L; *n* = 33) and the normal Hcy group (serum Hcy levels ≤15 μmol/L; *n* = 37). We compared the demographic and clinical characteristics of the two groups. As shown in Table 1, we found no differences in age, smoking status, alcohol use, and the prevalence of comorbidities—including hypertension, diabetes, coronary heart disease, anxiety/depression, malignancy, trauma, and autoimmune diseases—between the two groups (*p* > 0.05). The proportion of males and serum Hcy levels were higher, and folic acid levels were lower, in the high Hcy group than in the normal Hcy group (*p* < 0.05).

**Table 1:** Demographics and clinical characteristics of patients.

	All patients (*n* = 70)	High Hcy group (*n* = 33)	Normal Hcy group (*n* = 37)	*p*-value
Age at onset	26.64 ± 18.00	27.58 ± 20.17	25.81 ± 16.07	0.685
Sex, male, *n* (%)	34 (48.6)	21 (63.6)	13 (35.1)	0.017
Smoking, *n* (%)	8 (11.4)	3 (9.1)	5 (13.5)	0.714
Drinking, *n* (%)	4 (5.7)	1 (3.0)	3 (8.1)	0.616
Hypertension, *n* (%)	3 (4.3)	3 (9.1)	0	0.100
Diabetes, *n* (%)	1 (1.4)	0	1 (2.7)	1.000
Coronary heart disease, *n* (%)	3 (4.3)	2 (6.1)	1 (2.7)	0.599
Cerebrovascular disease, *n* (%)	0	0	0	1.000
Anxiety/depression, *n* (%)	2 (2.9)	1 (3.0)	1 (2.7)	1.000
Malignancy, *n* (%)	1 (1.4)	1 (3.0)	0	0.471
Trauma, *n* (%)	1 (1.4)	0	1 (2.7)	1.000
Autoimmune diseases, *n* (%)				
Sjogren syndrome	1 (1.4)	0	1 (2.7)	1.000
Thyroid disease	5 (7.1)	3(9.1)	2 (5.4)	0.661
Treatment, *n* (%)				
Corticosteroid	66 (94.3)	32 (97.0)	34 (92.0)	0.361
Intravenous immunoglobulin	14 (20)	6 (18.2)	8 (21.6)	0.719
Immunosuppressant	11 (15.7)	5 (15.2)	6 (16.2)	0.903
Azathioprine	4 (5.7)	1 (3.0)	3 (8.1)	0.616
Mycophenolate mofetil	6 (8.6)	3 (9.1)	3 (8.1)	0.661
Methotrexate	1 (1.4)	0	1 (2.7)	1.000
Initial EDSS, medians (interquartile ranges)	4 (3, 6)	4.5 (3.25, 6.5)	3.5 (2.5, 5)	0.038
Final EDSS, medians (interquartile ranges)	2 (1, 2.625)	2 (1, 3.75)	1 (0.5, 2)	0.015
Outcomes, poor recovery, *n* (%)	14 (20)	9 (27.3)	5 (13.5)	0.151
Follow-up interval (months)	17 (15, 26)	18 (15, 26)	16.5 (14, 28)	0.588
Relapse, *n* (%)	15 (21.4)	11 (33.3)	4 (10.8)	0.039

Data are presented as means ± standard deviations, numbers (percentages), or medians (interquartile ranges).

Hcy, homocysteine; EDSS, Expanded Disability Status Scale; IQR, interquartile range. *p < 0.05.

As shown in Table 2, no differences were found between the groups in intracranial pressure, proportion of brain lesions, optic nerve involvement, and spinal cord lesion segments, nor were differences found between the groups in levels of leukocytes, erythrocytes, hemoglobin, alanine aminotransferase, creatine, total cholesterol, triglycerides, high-density lipoprotein, low-density lipoprotein, cerebrospinal fluid leukocytes, or cerebrospinal fluid proteins (*p* > 0.05).

**Table 2:** Laboratory and imaging data of patients.

	All patients (*n* = 70)	High Hcy group (*n* = 33)	Normal Hcy group (*n* = 37)	*p*-value
Homocysteine levels (μmol/L)	15.61 ± 7.95	21.56 ± 7.77	10.29 ± 2.44	0.000
Folic acid (ng/mL)	7.51 ± 3.97	5.40 ± 2.96	9.40 ± 3.82	0.000
Vitamin B12 (pg/mL)	592.77 ± 393.35	516.20 ± 354.52	661.07 ± 417.94	0.125
Leukocyte counts, median (IQR) (×10^9^/L)	8.39 (6.51, 11.14)	8.26 (5.54, 10.96)	8.78 (6.73, 11.60)	0.960
Erythrocyte counts, median (IQR) (×10^12^/L)	4.38 (4.05, 4.75)	4.46 (4.11, 4.86)	4.29 (3.96, 4.58)	0.067
Hemoglobin, median (IQR) (g/L)	128 (118.08, 142.68)	128 (122.25, 142)	126 (117.5, 142.75)	0.743
ALT	15 (9, 25)	15 (9, 29.5)	14 (9, 21.5)	0.908
Creatine	55 (40.75,66.25)	58 (40, 67)	54 (43, 66)	0.953
Total cholesterol (mmol/L)	3.80 ± 0.96	3.76 ± 0.99	3.84 ± 0.94	0.714
Triglycerides (mmol/L)	1.24 ± 0.81	1.25 ± 0.58	1.23 ± 0.97	0.912
High-density lipoprotein (mmol/L)	1.19 ± 0.32	1.24 ± 0.35	1.15 ± 0.30	0.213
Low-density lipoprotein (mmol/L)	2.29 ± 0.71	2.30 ± 0.70	2.29 ± 0.73	0.961
Intracranial pressure (mmH_2_O)	160 (135, 180)	170 (14.5, 200)	155 (120, 175)	0.298
CSF leukocyte counts (×10^6^/L)	12 (4, 32)	14 (4, 36.5)	11 (4.5, 31)	0.985
CSF protein concentration (mg/L)	309.4 (234.55, 392.18)	310.2 (248.1, 384.9)	309 (226.2, 395.5)	0.489
Brain lesion, *n* (%)	48 (68.6)	21 (63.6)	27 (73.0)	0.401
Optic nerve involvement, *n* (%)	11 (15.7)	7 (21.2)	4 (10.8)	0.233
Spinal cord lesion segments, medians (interquartile ranges)	0 (0, 4.25)	0 (0, 7.5)	0 (0, 3.25)	0.521

Initial EDSS scores were higher in the high Hcy group than in the normal Hcy group (*p* = 0.038). Patients received different treatments, including corticosteroids, immunoglobulin, and immunosuppressants (azathioprine, mycophenolate mofetil, and methotrexate), according to their clinical symptoms and financial situations. There were no significant differences in these parameters between the groups.

### 3.2 Hcy levels were associated with poor clinical recovery in patients with first-attack MOGAD

**Figure 2:**
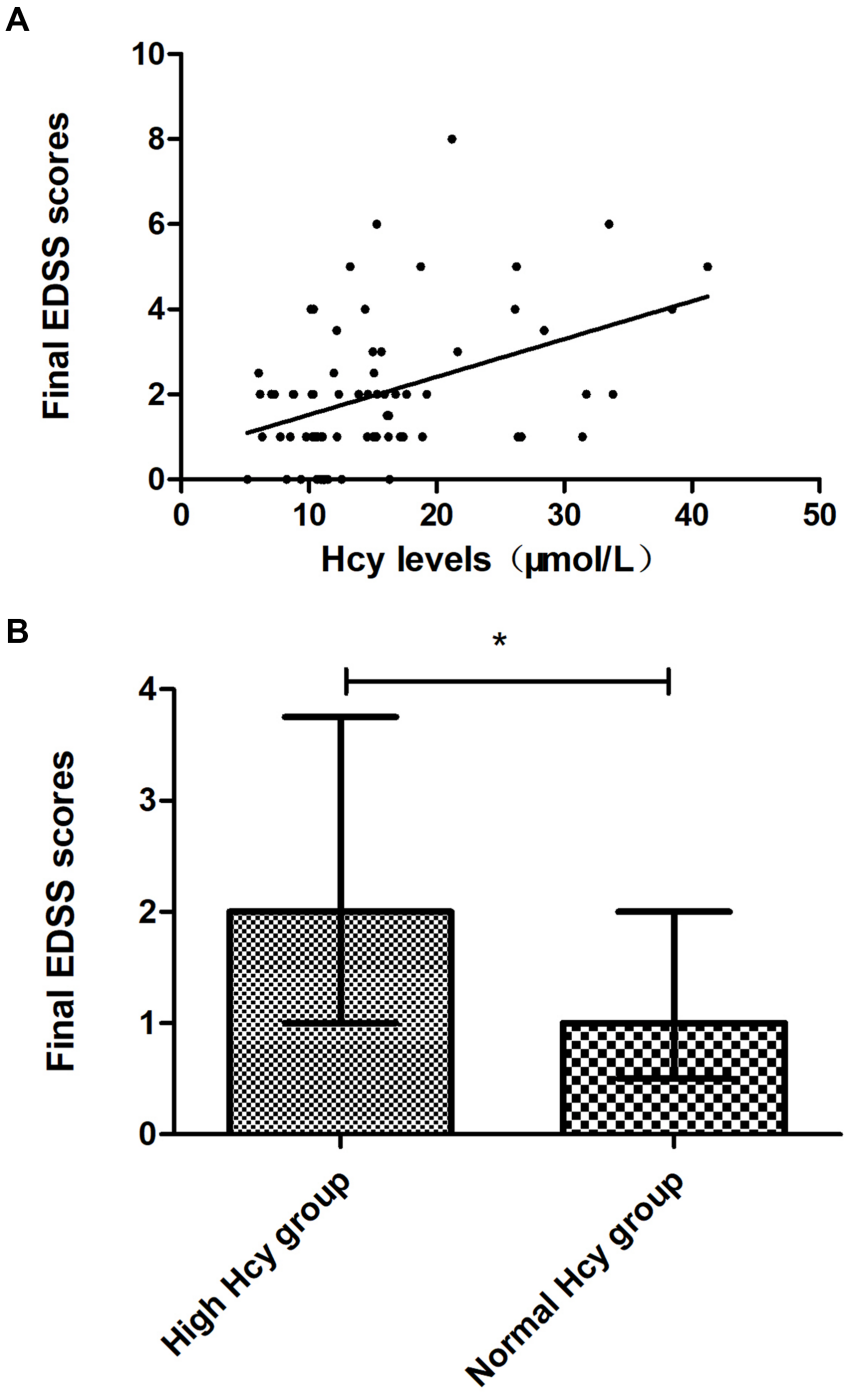
Association between Hcy levels and clinical recovery. **(A)** Final EDSS scores were higher in the high Hcy group than in the normal Hcy group (*p* < 0.05). **(B)** Hcy levels were positively correlated with the final EDSS scores (*p* < 0.05). EDSS, Expanded Disability Status Scale; Hcy, homocysteine. *p < 0.05.

We found that the final EDSS scores were higher in the high Hcy group than in the normal Hcy group, indicating higher levels of disability in the high Hcy group (*p* = 0.015) (Figure 2A). Univariate analysis indicated that serum Hcy levels [odds ratio (OR) 1.124, 95% confidence interval (CI) 1.042–1.212, *p* = 0.002] and initial EDSS scores (OR 1.873, 95% CI 1.320–2.658, *p* = 0.000) were associated with poor clinical recovery (Table 3). In the basic multivariate logistic regression analysis model, Hcy levels (OR 1.104, 95% CI 1.010–1.206, *p* = 0.029) and initial EDSS scores (OR 1.825, 95% CI 1.240–2.686, *p* = 0.002) were related to poor clinical recovery of MOGAD. In the adjusted model, Hcy levels (OR 1.126; 95% CI 1.005–1.261, *p* = 0.04) and initial EDSS scores (OR 2.017, 95% CI 1.266–3.214, *p* = 0.003) remained associated with poor clinical recovery of MOGAD (Table 4). With Spearman’s correlation analysis, we found that Hcy levels (*r* = 0.03397; *p* = 0.004) were positively correlated with final EDSS scores (Figure 2B).

**Table 3:** Univariate logistic regression analysis of potential risk factors that may predict poor clinical recovery in patients with first-attack MOGAD.

Variables	Univariate analysis OR (95% CI)	*p*-value
Age at onset	1.012 (0.98, 1.044)	0.474
Sex, male	3.333 (0.932, 11.924)	0.064
Initial EDSS	1.873 (1.320, 2.658)	0.000
Homocysteine levels	1.124 (1.042, 1.212)	0.002*
Folic acid	0.896 (0.754, 1.064)	0.209
Vitamin B12	1.000 (0.999, 1.002)	0.648

MOGAD, myelin oligodendrocyte glycoprotein antibody disease; OR, odds ratio; CI, confidence interval; EDSS, Expanded Disability Status Scale. ^*^p < 0.05.

**Table 4:** Multivariate logistic regression analysis of potential risk factors that may predict clinical recovery in patients with first-attack MOGAD.

Variables	Multivariate analysis			
	^a^Basic model		^b^Adjust I model	
	OR (95% CI)	*p*-value	OR (95% CI)	*p*-value
Age			1.006 (0.967, 1.046)	0.776
Sex, female	2.473 (0.541, 11.304)	0.243	3.024 (0568, 16.081)	0.194
Initial EDSS	1.825 (1.240, 2.686)	0.002	2.017 (1.266, 3.214)	0.003*
Homocysteine levels	1.104 (1.010, 1.206)	0.029	1.126 (1.005, 1.261)	0.04*
Folic acid			0.998 (0.782, 1.272)	0.984
Vitamin B12			1.002 (1.000, 1.004)	0.058

MOGAD, myelin oligodendrocyte glycoprotein antibody disease; OR, odds ratio; CI, confidence interval; EDSS, Expanded Disability Status Scale. ^*^p < 0.05.

a Basic model: variables with *p* < 0.1 in the univariate logistic regression analysis were included in the multivariate model.

b Adjusted I model: variables with *p* < 0.1 in the univariate logistic regression analysis or variables which may have an impact on the final EDSS score (including age at onset, sex, and initial EDSS score), and factors that affect Hcy levels (including folic acid and vitamin B12) were included in the adjusted model.

### 3.3 Association between Hcy levels and relapse in patients with first-attack MOGAD

There were 15 cases relapsed in the study. Among these relapsed cases, 6 cases presented with myelitis, 2 cases presented with optic neuritis, 5 cases presented with brain lesions and 2 cases presented with encephalomyelitis.

**Figure 3:**
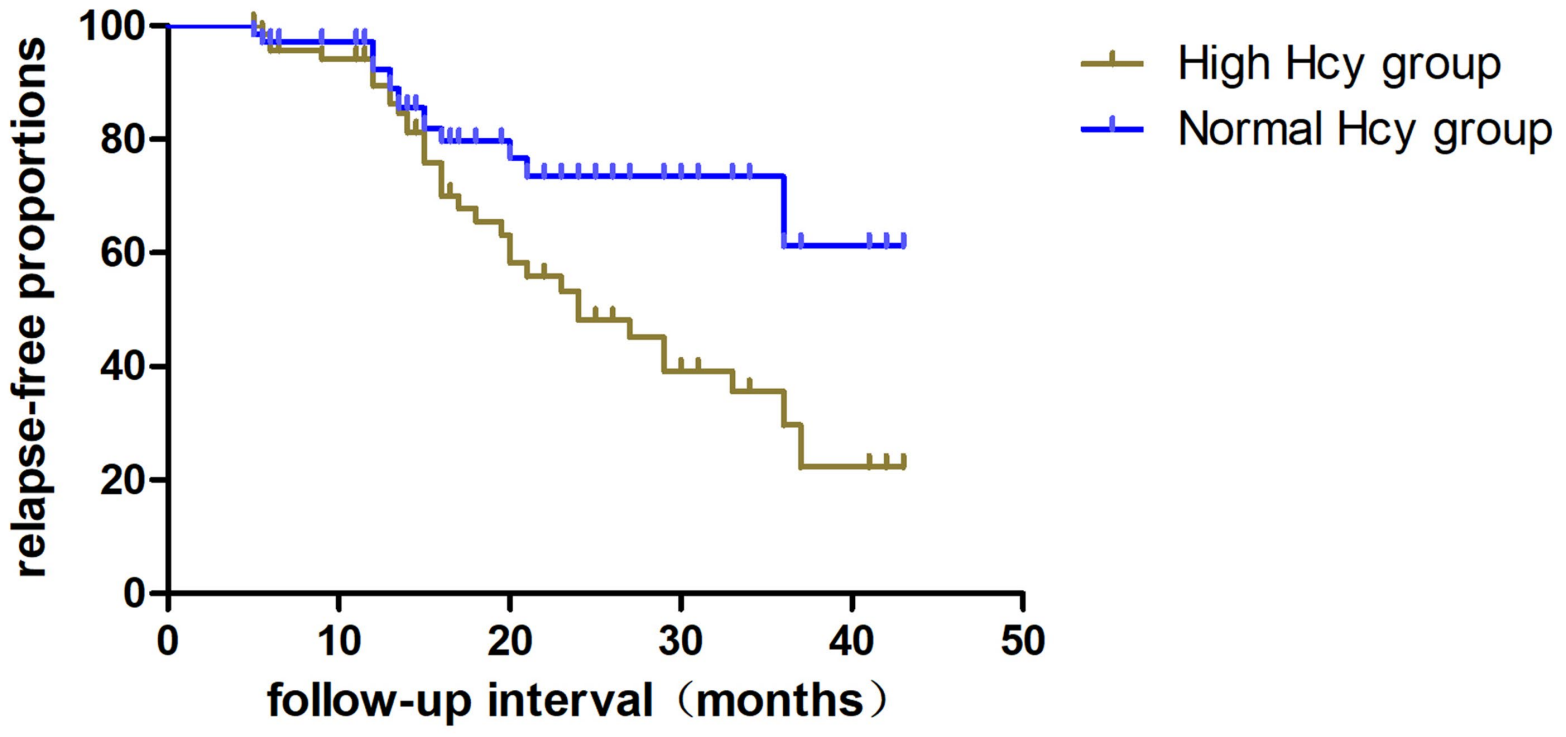
Kaplan–Meier analysis indicating the cumulative proportions of patients without relapse in the high Hcy group and normal Hcy group. Hcy, homocysteine.

The relapse rate was higher in the high Hcy group than in the normal Hcy group (*p* = 0.039) (Table 1). Kaplan–Meier survival analysis (Figure 3) showed that Hcy levels were a predictor of relapse in patients with MOGAD (log-rank test *p* = 0.029). Univariate analysis indicated that serum Hcy levels [hazard ratio (HR) 1.091, 95% CI 1.040–1.145, *p* = 0.000], age (HR 0.964, 95% CI 0.932–0.997, *p* = 0.034), and sex (HR 3.264, 95% CI 1.029–10.355, *p* = 0.045) were associated with the relapse of MOGAD (Table 5). In the basic multivariate Cox proportional hazards model, Hcy levels (HR 1.062, 95% CI 1.009–1.119, *p* = 0.022) were related to the relapse of MOGAD. In the adjusted model, Hcy levels (HR 1.088, 95% CI 1.020–1.161, *p* = 0.022) remained related to the relapse of MOGAD (Table 6), which indicated that Hcy levels may be a risk factor for predicting relapse.

**Table 5:** Univariate Cox proportional hazards models of potential risk factors that may predict relapse in patients with first-attack MOGAD.

Variables	Univariate analysis	
	HR (95% CI)	*p*-value
Age	0.964 (0.932, 0.997)	0.034
Sex, female	3.264 (1.029, 10.355)	0.045
Initial EDSS	0.905 (0.691, 1.186)	0.469
Homocysteine levels (μmol/L)	1.091 (1.040, 1.145)	0.000
Folic acid (ng/mL)	0.887 (0.768, 1.025)	0.104
Vitamin B12 (pg/mL)	0.999 (0.998, 1.001)	0.274

MOGAD, myelin oligodendrocyte glycoprotein antibody disease; HR, Hazard ratio; CI, confidence interval; EDSS, Expanded Disability Status Scale. ^*^p < 0.05.

**Table 6:** Multivariate logistic regression analysis of potential risk factors that may predict relapse in patients with first-attack MOGAD.

Variables	Multivariate analysis			
	^a^Basic model		^b^Adjust I model	
	HR (95% CI)	*p*-value	HR (95% CI)	*p*-value
Age	0.976 (0.942, 1.011)	0.173	0.975 (0.933, 1.018)	0.253
Sex, female	2.500 (0.756, 8.265)	0.133	2.187 (0.656, 7.0.283)	0.203
Initial EDSS			0.816 (0.586, 1.136)	0.229
Homocysteine levels	1.062 (1.009, 1.119)	0.022*	1.088 (1.020, 1.161)	0.011*
Folic acid			1.034 (0.882, 1.212)	0.681
Vitamin B12			0.999 (0.997, 1.001)	0.245

MOGAD, myelin oligodendrocyte glycoprotein antibody disease; HR, Hazard ratio; CI, confidence interval; EDSS, Expanded Disability Status Scale. ^*^p < 0.05.

a Basic model: variables with *p* < 0.1 in the univariate logistic regression analysis were included in the multivariate model.

b Adjust I model: variables with *p* < 0.1 in the univariate logistic regression analysis or variables which may have an impact on the relapse (including age at onset, sex and initial EDSS scores), and factors that affect Hcy levels (including folic acid and vitamin B12) were included in the adjusted model.

## 4 Discussion

MOGAD, a serious autoimmune disorder involving antibodies against MOG, causes high rates of disability and relapse, and predominantly affects the optic nerve and spinal cord (18). Even now, reliable predictors of MOGAD are limited. The present retrospective analysis explored a potential risk factor that may help predict the outcomes of patients with first-attack MOGAD. We found that patients in the high Hcy group experienced a worse recovery along with a higher relapse rate than those in the normal Hcy group. Therefore, Hcy levels may be a risk factor for predicting the prognosis and relapse of MOGAD. To exclude the effects of previous treatments—such as glucocorticoids, immunoglobulin, and immunosuppressants—and accurately calculate the relapse rate during the follow-up, we focused only on patients diagnosed with first-attack MOGAD.

The mean age at onset of MOGAD in this study was 26.64 years, consistent with that in previous reports (19, 20). Unlike the higher proportion of females reported among patients with NMOSD, the female-to-male ratio among our patients with MOGAD was approximately 1:1.

We defined hyperhomocysteinemia as a high Hcy (>15 μmol/L) level in the blood, in accordance with previous definitions (21). Based on this standard, we divided patients into either the high Hcy group or the normal Hcy group.

Hyperhomocysteinemia usually arises from genetic mutations of the enzymes involved in Hcy metabolism or from nutritional deficiencies of folate, vitamin B6, and vitamin B12 (22). Here, the folic acid levels were lower in the high Hcy group than in the normal Hcy group, which indicated that folic acid deficiency may increase the risk of hyperhomocysteinemia.

Many studies have investigated the association between Hcy and CNS neurodegenerative disorders and autoimmune demyelinating diseases (23, 24). Some have reported that patients with MS exhibit higher Hcy levels than healthy controls (10, 25). One previous study indicated that patients with MS with hyperhomocysteinemia showed a more advanced disease progression than patients with MS without hyperhomocysteinemia, demonstrating an association between elevated Hcy levels and disability (26). In our previous study, Hcy levels were identified as a predictor of both relapse and prognosis in patients with first-attack NMOSD (11). In addition, Hcy levels were significantly higher in patients with acute-phase NMOSD with an EDSS score ≥4 than in patients with an EDSS <4, which indicates that increased Hcy has the potential to affect the pathogenesis or progression of NMOSD (5). In the current study, we found that Hcy levels were an independent risk factor for predicting clinical recovery in patients with first-attack MOGAD and that Hcy levels were significantly associated with relapsing MOGAD.

The full impact of Hcy on the clinical outcomes of MOGAD is still not entirely clear. A previous study described a frequent association between MOGAD and NMDAR encephalitis. MOG antibodies were detected in 9/23 patients diagnosed with NMDAR encephalitis (27), indicating a relatively high overlap between NMDAR antibodies and MOGAD. Another study showed that 11.9% patients with MOGAD and 0.6% patients with NMOSD had overlapping NMDAR antibodies. MOGAD maybe more likely than NMOSD to co-exist with anti-NMDAR encephalitis. MOGAD may be more likely than NMOSD to co-exist with NMDARs. MOG antibodies could cause rapid *β*-tubulin dephosphorylation and disturb the cytoskeletal structure stability and microtubular polymerization of oligodendrocytes, thus leading to oligodendrocytopathy (28). Prior reports identified the existence of functional NMDARs on oligodendrocytes, which are activated under pathological conditions (29–32). Another study also showed NMDA receptor subunit expression on oligodendrocyte processes, and the presence of NMDA receptor subunit messenger RNA in isolated white matter (33).

Hcy is directly toxic to vascular endothelium. Hcy and its metabolites are excitatory agonists of the NMDAR. NMDA receptor activation resulted in rapid Ca2^+^-dependent detachment and disintegration of oligodendroglial processes in the white matter of mice expressing green fluorescent protein (GFP) specifically in oligodendrocytes. Besides, the overstimulation of NMDAR can cause excitotoxicity, increased production of free radicals, activation of caspases, and overloading of intracellular calcium, ultimately inducing neuronal injury and apoptosis (34, 35). Hcy may also induce structural instability and degeneration of the myelin sheath through inhibition of methyl donors, adversely affecting disease progression (7, 8). Furthermore, vitamin B12 and folate supplementation can decrease serum Hcy levels in patients with MS, improving both the physical and the mental aspects of their quality of life (36). We thus speculate that vitamin B12 and folate supplementation may contribute to better clinical outcomes in patients with MOGAD who have high Hcy levels.

This study has some limitations. First, the total number of patients was relatively small and from a single center. Second, the follow-up period was relatively short. Our results need to be further validated through larger, multicenter studies with longer follow-up periods. Last, the diet and genetic polymorphism may also influence homocysteine levels. However, as the study was a retrospective research, the diet and genetic polymorphisms data of some patients’ were incomplete, we will validate these data in further study.

## 5 Conclusion

We identified Hcy levels as an independent risk factor for predicting poor clinical recovery in patients with first-attack MOGAD. Hcy levels were also significantly associated with the relapse of MOGAD. The mechanism underlying the precise role of Hcy in MOGAD requires further research. Examining Hcy levels can be a potential prognostic marker for the early assessment of first-attack MOGAD and may allow for more individualized treatment plans.

## Data Availability

The datasets used and/or analyzed during the current study are available from the corresponding author on reasonable request.

## References

[1] Asseyer S Cooper G Paul F. Pain in NMOSD and MOGAD: a systematic literature review of pathophysiology, symptoms, and current treatment strategies. Front Neurol. (2020) 11:778. doi: 10.3389/fneur.2020.00778, PMID: 33473247 PMC7812141

[2] Fujihara K Cook LJ. Neuromyelitis optica spectrum disorders and myelin oligodendrocyte glycoprotein antibody-associated disease: current topics. Curr Opin Neurol. (2020) 33:300–8. doi: 10.1097/WCO.0000000000000828, PMID: 32374571

[3] Wynford-Thomas R Jacob A Tomassini V. Neurological update: MOG antibody disease. J Neurol. (2019) 266:1280–6. doi: 10.1007/s00415-018-9122-2, PMID: 30569382 PMC6469662

[4] Cobo-Calvo A Ruiz A Maillart E Audoin B Zephir H Bourre B. Clinical spectrum and prognostic value of CNS MOG autoimmunity in adults: the MOGADOR study. Neurology. (2018) 90:e1858–69. doi: 10.1212/WNL.0000000000005560, PMID: 29695592

[5] Liang J Liu J Fan R Chen Z Chen X Tong J. Plasma homocysteine level is associated with the expanded disability status scale in neuromyelitis optica spectrum disorder. Neuroimmunomodulation. (2019) 26:258–64. doi: 10.1159/000503426, PMID: 31655825

[6] Ho PI Ortiz D Rogers E Shea TB. Multiple aspects of homocysteine neurotoxicity: glutamate excitotoxicity, kinase hyperactivation and DNA damage. J Neurosci Res. (2002) 70:694–702. doi: 10.1002/jnr.10416, PMID: 12424737

[7] Darendelioglu E Aykutoglu G Tartik M Baydas G. Turkish propolis protects human endothelial cells in vitro from homocysteine-induced apoptosis. Acta Histochem. (2016) 118:369–76. doi: 10.1016/j.acthis.2016.03.007, PMID: 27085254

[8] Pan L Yin Y Chen J Ma Z Chen Y Deng X. Homocysteine, vitamin B12, and folate levels in patients with multiple sclerosis in Chinese population: a case-control study and meta-analysis. Mult Scler Relat Disord. (2019) 36:101395. doi: 10.1016/j.msard.2019.101395, PMID: 31521916

[9] Fan S Xu Y Ren H Guan H Feng F Gao X. Comparison of myelin oligodendrocyte glycoprotein (MOG)-antibody disease and AQP4-IgG-positive neuromyelitis optica spectrum disorder (NMOSD) when they co-exist with anti-NMDA (N-methyl-D-aspartate) receptor encephalitis. Mult Scler Relat Disord. (2018) 20:144–52. doi: 10.1016/j.msard.2018.01.007, PMID: 29414288

[10] Li X Yuan J Han J Hu W. Serum levels of homocysteine, vitamin B12 and folate in patients with multiple sclerosis: an updated meta-analysis. Int J Med Sci. (2020) 17:751–61. doi: 10.7150/ijms.42058, PMID: 32218697 PMC7085269

[11] Zhang J Li Y Zhou Y Zhao Y Xie H Duan R. Serum homocysteine level is a predictor of relapse and prognosis in patients with first-attack Neuromyelitis Optica Spectrum disorders. Front Neurol. (2021) 12:667651. doi: 10.3389/fneur.2021.667651, PMID: 34122309 PMC8187771

[12] Li Y Xie H Zhang J Zhou Y Jing L Yao Y. Clinical and radiological characteristics of children and adults with first-attack myelin oligodendrocyte glycoprotein antibody disease and analysis of risk factors for predicting the severity at disease onset in Central China. Front Immunol. (2021) 12:752557. doi: 10.3389/fimmu.2021.752557, PMID: 34975841 PMC8714638

[13] Banwell B Bennett JL Marignier R Kim HJ Brilot F Flanagan EP. Diagnosis of myelin oligodendrocyte glycoprotein antibody-associated disease: international MOGAD panel proposed criteria. Lancet Neurol. (2023) 22:268–82. doi: 10.1016/S1474-4422(22)00431-8, PMID: 36706773

[14] Panunzio MF Pisano A Antoniciello A Di Martino V Frisoli L Cipriani V. Supplementation with fruit and vegetable concentrate decreases plasma homocysteine levels in a dietary controlled trial. Nutr Res. (2003) 23:1221–8. doi: 10.1016/S0271-5317(03)00133-7

[15] Inojosa H Schriefer D Klöditz A Trentzsch K Ziemssen T. Balance testing in multiple sclerosis-improving neurological assessment with static Posturography? Front Neurol. (2020) 11:135. doi: 10.3389/fneur.2020.00135, PMID: 32174886 PMC7057229

[16] Kang SS Rosenson RS. Analytic approaches for the treatment of Hyperhomocysteinemia and its impact on vascular disease. Cardiovasc Drugs Ther. (2018) 32:233–40. doi: 10.1007/s10557-018-6790-1, PMID: 29679304

[17] Perła-Kaján J Jakubowski H. Dysregulation of epigenetic mechanisms of gene expression in the pathologies of Hyperhomocysteinemia. Int J Mol Sci. (2019) 20:3140. doi: 10.3390/ijms20133140, PMID: 31252610 PMC6651274

[18] Denève M Biotti D Patsoura S Ferrier M Meluchova Z Mahieu L. MRI features of demyelinating disease associated with anti-MOG antibodies in adults. J Neuroradiol. (2019) 46:312–8. doi: 10.1016/j.neurad.2019.06.001, PMID: 31228536

[19] Mariotto S Ferrari S Monaco S Benedetti MD Schanda K Alberti D. Clinical spectrum and IgG subclass analysis of anti-myelin oligodendrocyte glycoprotein antibody-associated syndromes: a multicenter study. J Neurol. (2017) 264:2420–30. doi: 10.1007/s00415-017-8635-4, PMID: 29063242 PMC5688213

[20] Cobo-Calvo Á Ruiz A D'Indy H Poulat AL Carneiro M Philippe N. MOG antibody-related disorders: common features and uncommon presentations. J Neurol. (2017) 264:1945–55. doi: 10.1007/s00415-017-8583-z, PMID: 28770374

[21] Stehouwer DA. Clinical relevance of hyperhomocysteinaemia in atherothrombotic disease. Drugs Aging. (2000) 16:251–60. doi: 10.2165/00002512-200016040-00001 10874520

[22] Curro M Gugliandolo A Gangemi C Risitano R Ientile R Caccamo D. Toxic effects of mildly elevated homocysteine concentrations in neuronal-like cells. Neurochem Res. (2014) 39:1485–95. doi: 10.1007/s11064-014-1338-7, PMID: 24867323

[23] Tawfik A Elsherbiny NM Zaidi Y Rajpurohit P. Homocysteine and age-related central nervous system diseases: role of inflammation. Int J Mol Sci. (2021) 22:6259. doi: 10.3390/ijms22126259, PMID: 34200792 PMC8230490

[24] Pi T Liu B Shi J. Abnormal homocysteine metabolism: an insight of Alzheimer's disease from DNA methylation. Behav Neurol. (2020) 2020:8438602. doi: 10.1155/2020/8438602, PMID: 32963633 PMC7495165

[25] Jamroz-Wiśniewska A Bełtowski J Bartosik-Psujek H Wójcicka G Rejdak K. Processes of plasma protein N-homocysteinylation in multiple sclerosis. Int J Neurosci. (2017) 127:709–15. doi: 10.1080/00207454.2016.1241782, PMID: 27671515

[26] Oliveira SR Flauzino T Sabino BS Kallaur AP Alfieri DF Kaimen-Maciel DR. Elevated plasma homocysteine levels are associated with disability progression in patients with multiple sclerosis. Metab Brain Dis. (2018) 33:1393–9. doi: 10.1007/s11011-018-0224-4, PMID: 29797117

[27] Titulaer MJ Höftberger R Iizuka T Leypoldt F McCracken L Cellucci T. Overlapping demyelinating syndromes and anti–N-methyl-D-aspartate receptor encephalitis. Ann Neurol. (2014) 75:411–28. doi: 10.1002/ana.24117, PMID: 24700511 PMC4016175

[28] Dale RC Tantsis EM Merheb V Kumaran RY Sinmaz N Pathmanandavel K. Antibodies to MOG have a demyelination phenotype and affect oligodendrocyte cytoskeleton. Neurol Neuroimmunol Neuroinflamm. (2014) 1:e12. doi: 10.1212/NXI.0000000000000012, PMID: 25340056 PMC4202678

[29] Lipton SA. NMDA receptors, glial cells, and clinical medicine. Neuron. (2006) 50:9–11. doi: 10.1016/j.neuron.2006.03.026, PMID: 16600850

[30] Matute C. Oligodendrocyte NMDA receptors: a novel therapeutic target. Trends Mol Med. (2006) 12:289–92. doi: 10.1016/j.molmed.2006.05.004, PMID: 16750425

[31] Verkhratsky A Kirchhoff F. NMDA receptors in glia. Neuroscientist. (2007) 13:28–37. doi: 10.1177/1073858406294270, PMID: 17229973

[32] Wong R. NMDA receptors expressed in oligodendrocytes. BioEssays. (2006) 28:460–4. doi: 10.1002/bies.20402, PMID: 16615088

[33] Salter MG Fern R. NMDA receptors are expressed in developing oligodendrocyte processes and mediate injury. Nature. (2005) 438:1167–71. doi: 10.1038/nature04301, PMID: 16372012

[34] Drachtman RA Cole PD Golden CB James SJ Melnyk S Aisner J. Dextromethorphan is effective in the treatment of subacute methotrexate neurotoxicity. Pediatr Hematol Oncol. (2002) 19:319–27. doi: 10.1080/08880010290057336, PMID: 12078863

[35] Sibarov DA Abushik PA Giniatullin R Antonov SM. GluN2A subunit-containing NMDA receptors are the preferential neuronal targets of homocysteine. Front Cell Neurosci. (2016) 10:246. doi: 10.3389/fncel.2016.00246, PMID: 27847466 PMC5088185

[36] Nozari E Ghavamzadeh S Razazian N. The effect of vitamin B12 and folic acid supplementation on serum homocysteine, Anemia status and quality of life of patients with multiple sclerosis. Clin Nutr Res. (2019) 8:36–45. doi: 10.7762/cnr.2019.8.1.36, PMID: 30746346 PMC6355946

